# Noninvasive Disease Assessment in Eosinophilic Esophagitis With Fractionated Exhaled Nitric Oxide, Blood, and Fecal Biomarkers

**DOI:** 10.1097/MCG.0000000000002068

**Published:** 2024-09-02

**Authors:** Andreas Göldi, Tanay Kaymak, Luca Esposito, Anouk Lehmann, Simona Negoias, Michael Tamm, Jan Hendrik Niess, Petr Hruz

**Affiliations:** * University Digestive Healthcare Center, Clarunis; † Department of Otorhinolaryngology, University Hospital of Basel; ‡ Clinic of Respiratory Medicine, University Hospital Basel, Basel, Switzerland

**Keywords:** eosinophilic esophagitis, biomarker, FeNO, EDN, ECP

## Abstract

**Background:**

Eosinophilic Esophagitis (EoE) is a chronic inflammatory condition of the esophagus triggered by food and aeroallergens. There is a need for noninvasive biomarkers that reliably detect EoE in patients with cardinal symptoms and predict treatment response to reduce endoscopic evaluations.

**Study:**

Nonasthmatic patients 18 years or above with suspected or diagnosed EoE, gastroesophageal reflux disease (GERD), and control individuals with indication for endoscopy were enrolled prospectively between November 2020 and May 2022. Participants underwent body plethysmography with fractionated exhaled nitric oxide (FeNO) level measurement. Besides, serum and fecal biomarkers were measured by ELISA. A follow-up examination was scheduled after treatment initiation in patients with active EoE.

**Results:**

The median FeNO level in active EoE (20 ppb) was higher compared with GERD (15 ppb, *P*=0.038) and control individuals (14 ppb, *P*=0.046). Median FeNO did not significantly differ in EoE patients who underwent follow-up assessment after treatment response (20 ppb vs. 18 ppb, *P*=0.771). Serum EDN, ECP, and the absolute eosinophil blood count (AEC) were elevated in active EoE compared with control individuals but not compared with GERD except for AEC. Serum EDN, ECP and AEC decreased in EoE in remission at follow-up assessment. None of the fecal biomarkers was elevated in active EoE or during treatment.

**Conclusions:**

Assessment of FeNO may have diagnostic value in differentiating patients with active EoE from non-EoE patients but is not a suitable marker for monitoring disease activity. Serum EDN, ECP, TARC, and AEC levels are emerging as potential candidates for monitoring disease activity in EoE.

Eosinophilic Esophagitis (EoE) is a chronic type 2 immune-mediated inflammatory disease of the esophagus due to food and aeroallergen exposure leading to inflammation.^1,2^ First described barely 3 decades ago, EoE has emerged steadily, especially in Western Europe, with an escalating prevalence of up to 50 patients per 100,000 inhabitants.^3–6^ Cardinal symptoms in adults with EoE are solid food dysphagia, food impaction requiring endoscopic removal, or slowed food intake leading to a significant reduction in quality of life.^1,2^ EoE is characterized by an eosinophil-predominant inflammation with infiltration of ≥15 eosinophils per high power field (eos/hpf)^7,8^ and is associated with atopic comorbidities like asthma, rhinitis, or atopic dermatitis.^9,10^ Endoscopic signs and severity of symptoms may not always correspond with inflammatory disease activity, providing challenges in EoE diagnosis and monitoring after treatment initiation.^11,12^ According to current guidelines, repeated esophagogastroduodenoscopies (EGD) with esophageal tissue sampling are required to diagnose EoE and assess treatment response.^7^ To date, biomarkers in serum, plasma, urine, or feces are not used in the clinical routine.^13,14^ The esophageal string test is discussed as an alternative to EGD. Still, its usefulness as a less invasive test has to be further validated in the clinic.^15^ Thus, studies identifying suitable biomarkers address an essential avenue in EoE research to minimize invasive procedures required for the care of EoE.^16,17^

Fractionated exhaled nitric oxide (FeNO) could be a potential marker as FeNO is being used to monitor type 2 airway inflammation in atopic asthma, eosinophil-predominant asthma, bronchitis, chronic rhinosinusitis, or nasal polyposis.^18–22^ Nitric oxide (NO) is produced in the airways and can be measured by a nitric oxide analyzer in the exhaled air. NO is crucial in predominantly atopic airway inflammation as a mediator and regulator of lower airway inflammation.^23^ Inducible nitric oxide synthase (iNOS), responsible for upregulating NO, is also elevated in gastrointestinal diseases such as Inflammatory Bowel Disease (IBD).^24–26^ Therefore, we hypothesized that increased FeNO may also be observed in patients with active EoE.

Besides FeNO values, we aimed to examine other potential noninvasive biomarkers in the serum and the feces to support the diagnosis and monitoring of EoE disease activity either as a single marker or in combination with FeNO. For this purpose, markers of eosinophil activation and migration are plausible targets. However, to this day, no single noninvasive marker in blood and feces or semi-invasive marker collected from esophageal string brushing has been validated for routine clinical use.^16,17^ The absolute eosinophil blood count (AEC), eosinophil-derived neurotoxin (EDN), eosinophil cationic protein (ECP), major basic protein 1 (MBP-1), eotaxin 3, galectin-10, and thymus and activation-regulated chemokine (TARC) seem to be promising targets besides various other cytokines (eg, interleukin 4, 5, 13, and 33) because of their involvement in the EoE pathogenesis.^8,15,16,27,28^ Fecal calprotectin (fCal) is produced by neutrophils in the gut and plays a significant role as a marker in IBD and other lower tract gastrointestinal diseases.^29,30^ In EoE, however, fCal derivation is unclear so far and therefore we hypothesized fCal may be a plausible marker to examine.

In this study, we aimed to assess the utility of FeNO in differentiating patients with active EoE from gastroesophageal reflux disease (GERD) and from control individuals presenting without endoscopic and histologic signs of esophageal inflammation, as well as to monitor and predict EoE disease activity during treatment. Moreover, selected serum and fecal eosinophil biomarkers were evaluated to assess their ability to discriminate EoE from GERD and control individuals, either as a single marker or in combination with FeNO.

## MATERIALS AND METHODS

### Study Design and Study Population

We conducted a prospective, observational cohort study from November 2020 to May 2022 at the University Digestive Healthcare Center, Clarunis, Basel, Switzerland, with the inclusion of patients with suspected or diagnosed EoE presenting with dysphagia, heartburn, or food impaction.

Eligible patients aged 18 years or above were approached consecutively for study participation within a week before the planned EGD. Recruited patients who showed no pathology in the endoscopic evaluation of the esophagus and did not fulfill the clinical and histopathologic criteria for EoE diagnosis served as controls. These control individuals, however, are not seen as completely healthy subjects since they suffered from symptoms leading to EGD evaluation and other upper gastrointestinal diseases such as functional dyspepsia or gastritis could be an underlying cause. Control patients did not fulfill the clinical and endoscopic criteria for further evaluation for a motility disorder. Furthermore, absence of characteristic endoscopic and histopathologic signs for esophagitis excludes EoE variants as underlying cause of symptoms. Symptomatic individuals showing endoscopic signs of GERD according to the Los Angeles classification were included in the study cohort to differentiate between active EoE and GERD.

### Participant Eligibility

Participating individuals were excluded from assessment when they suffered from known physician-diagnosed asthma or frequently used inhalers. In cases of uncertain asthma diagnosis or asthma symptoms in the past (eg, childhood, history of a unique asthma episode, not-physician-proven asthma) with no current evidence for asthma and lack of asthma symptoms at the time of inclusion, patients were evaluated with a lung function assessment and body plethysmography. They were excluded from biomarker analysis if they suffered from an active obstruction defined by an FEV1/FVC <70% indicative of asthma or other obstructive lung diseases, which could potentially interfere with the assessment of FeNO levels. Furthermore, patients were excluded from fecal biomarker analysis if *Helicobacter pylori* presence was confirmed in gastric biopsies obtained during EGD.

Patients with concomitant chronic inflammatory gastrointestinal diseases such as IBD, current or past malignant disease, systemic radiation- or chemotherapy, or hypereosinophilic syndrome (peripheral blood eosinophils >1.5×10^9^/L) were excluded.

### Study Procedures

Routine gastroscopy and endoscopic evaluation for signs of EoE using the modified EoE endoscopic reference score (mEREFS) and routine esophageal tissue sampling were performed by gastroenterologists.^31^ A board-certified pathologist assessed the eosinophil numbers per high power field (eos/hpf) upon histologic examination for EoE. EoE was diagnosed according to the current clinical and histologic guidelines by the study participant’s treating gastroenterologist. Patients’ medical data were obtained from the hospital’s clinical patient data system.

Routine lung function test consisted of whole-body plethysmography with diffusion capacity and measurement of FeNO levels. These investigations were conducted in the Department of Pneumology, and a board-certified pneumologist evaluated the results. A routine nitric oxide analyzer from Stallargenes and Greer (Zurich, Switzerland) was used to measure FeNO levels. FeNO measurement was performed before EGD on the same day or within a few days before the gastroscopy appointment.

Patients with newly diagnosed EoE or known EoE presenting with ≥15 eos/hpf at the time of inclusion were scheduled for a follow-up assessment (including EGD and FeNO measurement), which was conducted at least 6 weeks after treatment initiation.

Venous blood was routinely drawn on the same day before the gastroscopy. The patient’s blood was kept at room temperature for 30 minutes and was then centrifuged at 2000*g* for 10 minutes. After centrifugation, serum was collected, aliquoted, and frozen at −80 °C until biomarker analysis.

The patients collected stool specimens 24 hours before the gastroscopy in a sterile stool collection tube sent to the patients before the appointment. If patients failed to bring the stool specimen on the day of the gastroscopy, the first stool sample after gastroscopy was collected and sent to our department within 72 hours. Stool samples collected or arrived later were excluded from analysis since values may be false—high due to biopsy sampling and possible micro bleedings from the EGD procedure. Upon arrival, stool samples were put on ice and immediately aliquoted into sterile tubes in portions of 50mg and were stored at −80 °C until biomarker measurements.

### Blood and Fecal Biomarker Analysis

AEC was determined via the Hospital Laboratory System. Quantification of serum and fecal biomarkers was conducted in the Gastroenterology Research Laboratory of our hospital. According to the manufacturer’s instructions, samples were thawed only once and measured by Enzyme-linked Immunosorbent Assays (ELISA) in duplicates on 96-well plates. Potential serum and fecal biomarkers were measured using the following ELISA kits: EDN (Immunodiagnostic AG), ECP (Aviscera Bioscience), MBP-1 (Cloud Clone), TARC (R&D Systems). Fecal calprotectin quantification was determined by Bühlmann AG (Schönenbuch, Switzerland) with an immunoturbidimetric method using a Mindray BS-380 analyzer.

In a preanalysis, promising biomarkers were determined in 40 samples using ELISA but were discarded from definitive analysis due to lack of detectability. Therefore, galectin-10 (Novus Biologicals), interleukin (IL)-23, eotaxin-3, IL-9 (R&D Systems), IL-4, 5, 9, 12p70, 13, 17, thymic stromal lymphopoietin (TSLP), periostin and kallikrein 5 (Luminex multiplex Assay from R&D Systems) were not further evaluated.

### Statistical Analysis

Data with nonparametric distribution are shown as median with interquartile range (IQR) and as mean with SD for parametric variables if not otherwise specified. For group comparisons, the Whitney Test, Wilcoxon rank-sum test, unpaired and paired *t* test, and ANOVA Kruskal-Wallis-test for multiple group comparison were used according to normality distribution. Correlations were calculated by using Spearman rank correlation and simple linear regression lines. Statistical analysis was performed using Prism Version 9.4.1 (GraphPad Software). *P-*values <0.05 were considered statistically significant.

### Ethic Disclosure

All participants provided written informed consent for participation before gastroscopy and any study procedures. The study was approved by the local ethics committee of Northwest and Central Switzerland (EKNZ; Project-ID 2019-00273).

## RESULTS

### Baseline Characteristics

**FIGURE 1:**
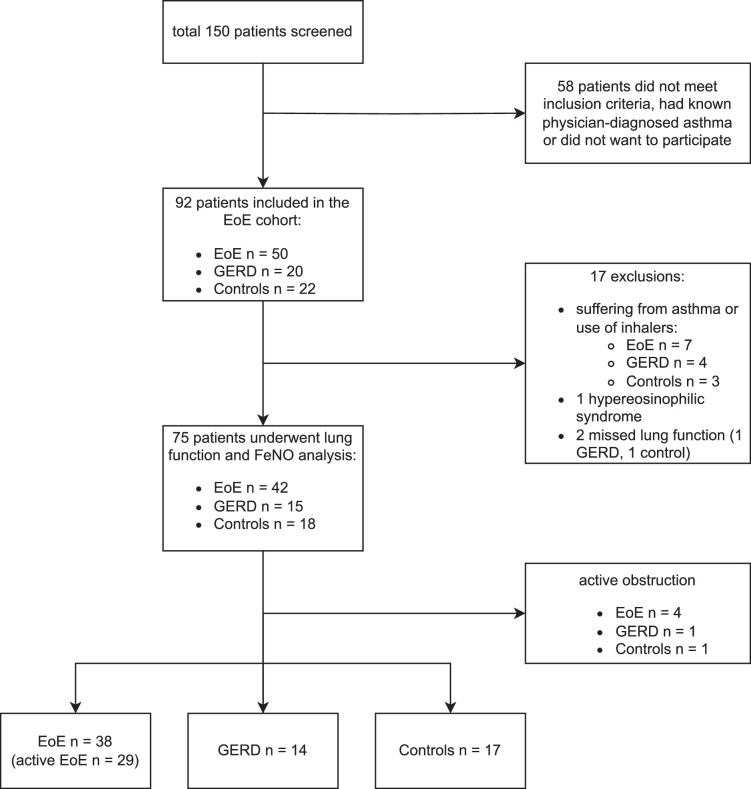
Flowchart of patient participation for body plethysmography and FeNO biomarker assessment. Among EoE patients, 29 patients presented with active EoE and 9 patients with EoE in remission. Of those with active EoE, 18 patients underwent follow-up assessment. GERD patients (n=14) and control individuals (n=17) scheduled for a routine gastroscopy served as control groups. FeNO indicates fractionated exhaled nitric oxide.

Out of 150 initially screened patients, 92 were assessed for eligibility to be enrolled in our study (Fig. 1). Of these, 17 patients did not meet the criteria for lung function testing, mainly due to asthma or regular use of physician-prescribed inhalers. The remaining 75 patients were eligible for biomarker analysis and body plethysmography with measurement of FeNO levels. Of those, 4 patients with EoE, 1 with GERD, and 1 control individual showed an active obstruction (FEV1/FVC <70%) indicative of concomitant asthma or another obstructive lung disease and were thus excluded from the final analysis.

In total, FeNO, serum, and fecal biomarker levels were analyzed in 69 patients, of whom 38 suffered from EoE. 29 patients presented with active EoE, of which 18 patients underwent repeated FeNO measurement as follow-up evaluation during treatment. Nine patients with known EoE were in remission at the initial gastroscopy and were analyzed alongside 11 patients in remission at follow-up. In addition, 14 patients with GERD and 17 control individuals were evaluated. The mean age in the EoE group was 41.8 years and did not differ significantly from patients with GERD or control individuals. Patients with EoE were 73.7% male, the median time since first EoE symptoms was 7 years, and the median diagnostic delay counted 3.5 years. The most frequent symptoms leading to gastroscopy in patients with EoE included dysphagia (94.7%), a history of food impaction requiring endoscopic removal (84.2%), and heartburn (42.1%). Detailed patient characteristics are shown in Table 1.

**TABLE 1:** Baseline Characteristics of Patients With EoE, GERD and Control Individuals

Patient characteristics	EoE n=38	GERD n=14	Controls n=17
Age, mean±SD	41.8±13.9	42.6±16.1	42.1±9.83
Male sex, n (%)	28 (73.7)	9 (64.3)	9 (52.9)
White race, n (%)	33 (86.8)	12 (85.7)	15 (88.3)
BMI, kg/m^2^, mean±SD	25.9±4.55	28.3±3.43	26±4.65
Time since first EoE symptoms, y median±IQR	7±9	—	—
Time since first EoE diagnosis, y median±IQR	2±6.25	—	—
Time between first symptoms and diagnosis, y median±IQR	3.5±7.5	—	—
History of esophageal dilatations, n (%)	4 (10.5)	0	0
History of atopic diseases, n (%)	22 (57.9)	2 (14.3)	4 (23.5)
rhinitis or rhinoconjunctivitis, n (%)	21 (55.3)	2 (14.3)	4 (23.5)
atopic dermatitis, n (%)	3 (7.9)	0	0
food allergies, n (%)	7 (18.4)	0	1 (5.9)
Smoker, active n (%)	7 (18.4)	8 (57.1)	4 (23.5)
occasional smoker, n (%)	6 (85.7)	2 (25)	2 (50)
former smoking, n (%)	10 (26.3)	1 (7.1)	5 (29.4)
pack years, median±IQR	0±2.25	2.5±7.5	1±11
Symptoms experienced, n (%)			
Dysphagia	36 (94.7)	5 (35.7)	7 (41.2)
Odynophagia	7 (18.4)	3 (21.4)	1 (5.9)
Solid food impaction	32 (84.2)	1 (7.14)	0 (0)
Retrosternal pain	12 (31.6)	5 (35.7)	4 (23.5)
Heartburn	16 (42.1)	11 (78.6)	9 (52.9)
Nausea and vomitus	4 (10.5)	2 (14.3)	1 (5.9)
Treatment, n (%)			
PPI	15 (39.5)	2 (14.3)	2 (11.8)
Topical steroids	9 (23.7)	0	0
Diet	2 (5.3)	0	0
None	17 (44.7)	14 (85.7)	15 (88.2)
Endoscopic signs suggestive of EoE, n (%)			
Edema	11 (29)	—	—
White exudates	16 (42.1)	—	—
Longitudinal furrows	24 (63.2)	—	—
Rings	17 (44.7)	—	—
Stricture	5 (13.2)	—	—
Crepe paper esophagus	3 (7.9)	—	—
Total mEREFS score, median±IQR	2±3	—	—

EoE (n=38) active at baseline n=29, EoE in remission at baseline n=9.

BMI indicates body mass index; GERD, gastroesophageal reflux disease; IQR, interquartile range; mEREFS, modified endoscopic reference score; PPI, proton pump inhibitor.

### Elevated FeNO and AEC in Patients With Active EoE

Mean forced expiratory flow in the first second of forced expiration (FEV1) and the measured Tiffeneau index indicating obstructive lung disease did not differ between all 3 study groups (Supplemental Table 1, Supplemental Digital Content 1, http://links.lww.com/JCG/B129).

**FIGURE 2:**
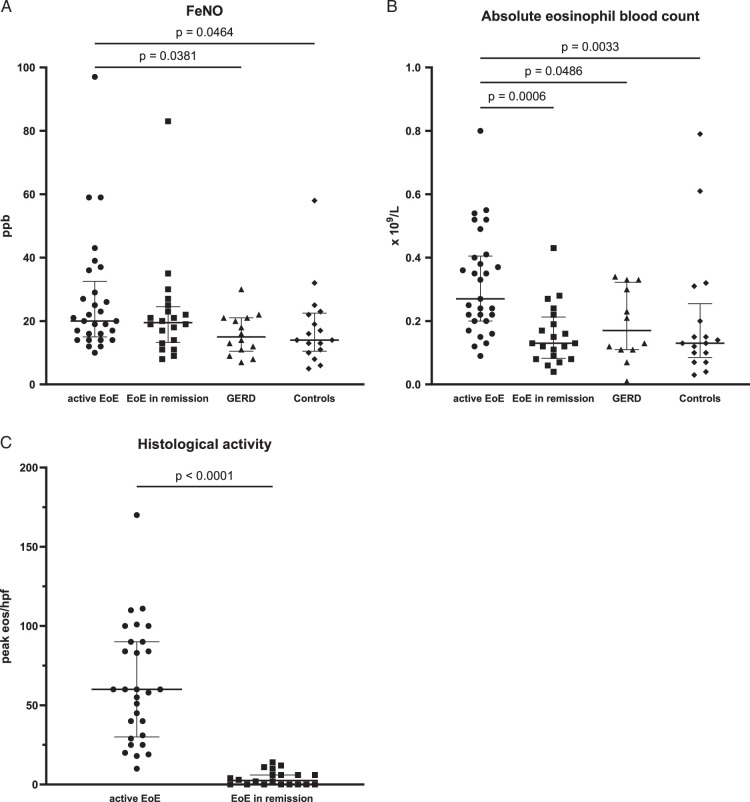
FeNO, AEC biomarker, and histologic activity assessment in EoE, GERD and control individuals in nonasthmatic subjects. Bars show median and IQR. Active EoE (n=29), EoE in remission (n=20) (including 11 patients from follow-up with successful treatment), GERD (n=14), controls (n=17). A, FeNO values in ppb. B, Absolute eosinophil blood count (AEC) in active EoE versus EoE in remission, GERD, and controls. C, Peak eosinophil count per high power field in esophageal biopsies of patients with active EoE compared with EoE in remission (Mann-Whitney test *P*<0.0001).

The median FeNO level in active EoE was 20 ppb (IQR 17.5 ppb), ranging from 10 to 97 ppb (Table 2 and Fig. 2A). Compared with patients with active EoE, the median FeNO in GERD patients and control individuals was significantly lower with 15 ppb (IQR 10.5 ppb, *P*=0.038) and 14 ppb (IQR 12 ppb, *P*=0.046), respectively. However, no difference was observed when comparing FeNO in patients with active EoE versus remission. Interestingly, more patients with active EoE, 37.9% (11/29), have FeNO values >25ppb than patients in remission, 25% (5/20), control individuals, 17.7% (3/17), or GERD patients, with only 7.1% (1/14).

**TABLE 2:** FeNO and Serum Biomarker Levels in Patients With EoE and GERD and in Control Individuals

Biomarker	Active EoE	EoE in remission	GERD	Controls	*P*
FeNO (ppb), median (IQR)	20 (17.5)	19.5 (11.2)	15 (10.5)	14 (12)	**0.044**
Eos/hpf, no, median (IQR)	60 (60)	2.5 (6)	—	—	**<0.0001**
AEC (x10^9^/L), median (IQR)	0.27 (0.205)	0.13 (0.13)	0.17 (0.213)	0.13 (0.17)	**0.0004**
EDN serum (ng/mL), median (IQR)	72.36 (45.35)	23.41 (42.33)	42.45 (77.71)	26.4 (41.64)	**0.004**
ECP serum (ng/mL), median (IQR)	112.3 (116.35)	76.65 (76.57)	136 (100.55)	56.3 (41)	**0.015**
TARC serum (ng/mL), mean (SD)	833.2 (464.5)	807.5 (534.7)	1049 (505.8)	783.3 (411.9)	0.434
MBP-1 serum (ng/mL), mean (SD)	526.2 (202.4)	569.2 (284)	574.7 (174.6)	464.5 (212.8)	0.465

Bold value represent the *P*-value.

*P-*values result from Kruskal-Wallis or ordinary ANOVA test comparing active EoE versus GERD and control individuals according to normality distribution.

AEC indicates absolute eosinophil blood count; ECP, eosinophil cationic protein; EDN, eosinophil-derived neurotoxin; eos/hpf, eosinophil per high power field; FeNO, fractionated exhaled nitric oxide; MBP-1, major basic protein 1; ppb, parts per billion; TARC, thymus and activation-regulated chemokine.

We performed a ROC analysis to determine the predictability of active EoE versus non-EoE patients (GERD and control individuals) using a cutoff level for FeNO of >15 and >25.5 ppb. The cutoff value of >15 ppb demonstrated good sensitivity (75.9%) for detecting active EoE in our cohort but poor specificity (51.6%). Conversely, a FeNO cutoff level of >25.5 ppb resulted in reasonable specificity (90.3%) but poor sensitivity (34.5%; Supplemental Fig. 1A, Supplemental Digital Content 1, http://links.lww.com/JCG/B129).

Interestingly, the analysis of the absolute peripheral eosinophil blood count revealed that patients with active EoE had significantly higher median AEC (0.27 ×10^9^/L, IQR 0.205) compared with control individuals (0.13×10^9^/L, IQR 0.17, *P*=0.003) and GERD patients (0.17×10^9^/L, IQR 0.213, *P*=0.049, Fig. 2B) and also EoE patients in remission (0.13 ×10^9^/L, IQR 0.13, *P*=0.0006). This finding is consistent with the histologic analysis in active EoE compared with EoE patients in remission (median of 60 eos/hpf vs. 2.5 eos/hpf, *P*<0.0001, Fig. 2C). In a ROC analysis, an AEC cutoff of 0.345×10^9^/L demonstrated high specificity (93.1%) but low sensitivity (44.8%) in discriminating active EoE patients from non-EoE individuals. A cutoff value of 0.215×10^9^/L exhibited modest sensitivity (72.4%) and specificity (68.9%; Supplemental Fig. 1B, Supplemental Digital Content 1, http://links.lww.com/JCG/B129).

### AEC But Not FeNO Decreases in EoE Treatment Responders

**FIGURE 3:**
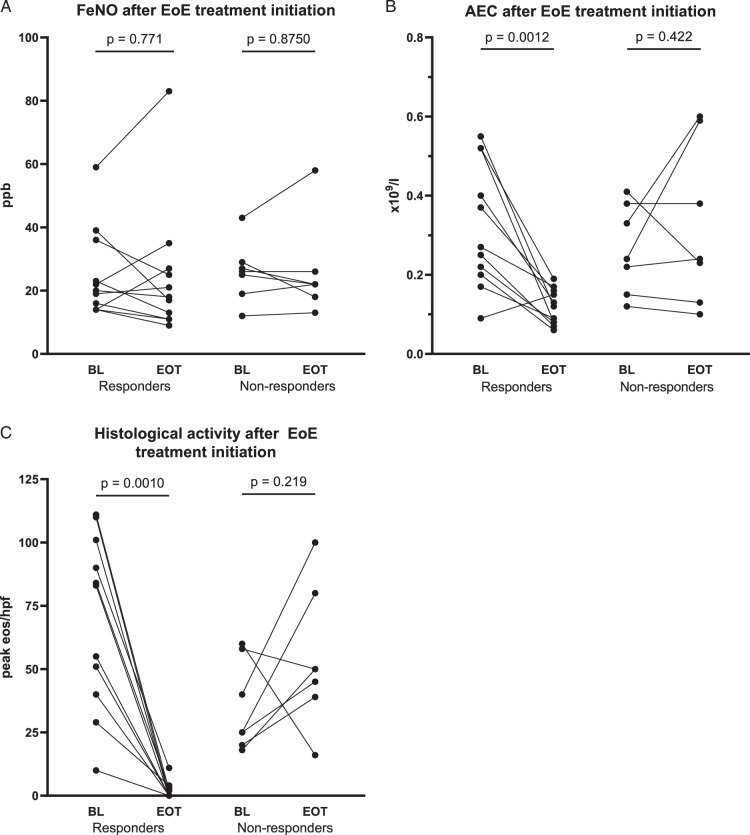
FeNO and AEC biomarker levels at baseline (BL) and after treatment initiation (EOT) in patients with EoE were shown for treatment responders and nonresponders. A, FeNO levels in responders and nonresponders (Wilcoxon test). B, AEC levels in responders and nonresponders (paired *t* test). C, Peak eos/hpf in responders and nonresponders (Wilcoxon test).

Lung function testing with FeNO assessment was repeated in 18 patients with active EoE during topical steroid treatment, proton pump inhibitors (PPI), or dietary therapy. At the time of the second EGD, 11 patients were in clinical and histologic remission, and 7 had persisting active disease despite treatment. No significant decrease in FeNO levels was observed in responders (median 20 ppb vs. 18 ppb, *P*=0.771) and nonresponders (median 26 ppb vs. 22 ppb, *P*=0.875) at follow-up, compared with baseline (Fig. 3A).

However, the treatment responders exhibited a significant decrease in absolute eosinophil blood count and achieved histologic remission. Pretreatment mean AEC of 0.324×10^9^/L decreased to 0.118×10^9^/L (*P*=0.001, Fig. 3B) at follow-up. In nonresponders, no difference in mean AEC levels was observed before and during treatment (mean AEC pretreatment 0.264×10^9^/L vs. mean AEC follow-up 0.324×10^9^/L, *P*=0.422). All in line with a decrease in peak mucosal eosinophil infiltration in responders [pretreatment median 83 eos/hpf vs. follow-up 0 eos/hpf (*P*<0.001)], compared with a nonsignificant increase of esophageal eosinophilia in nonresponders (pretreatment median 25 eos/hpf vs. median follow-up 50 eos/hpf, *P*=0.219, Fig. 3C).

### Elevated EDN in EoE With Decrease in Treatment Responders

**FIGURE 4:**
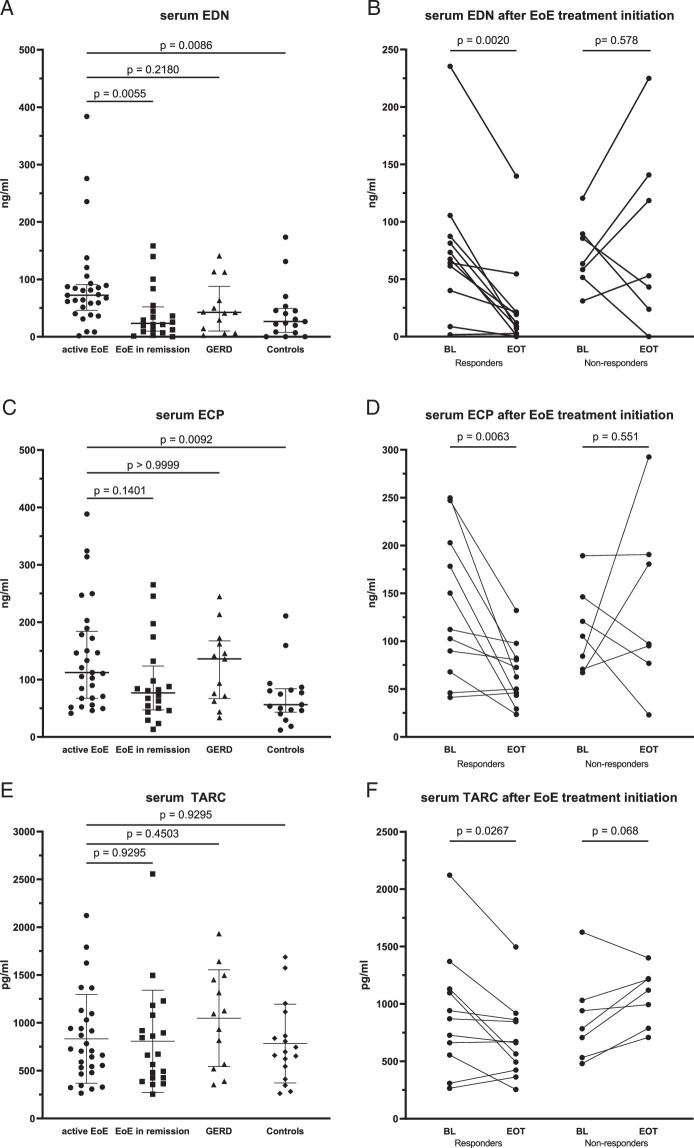
Serum biomarker levels in EoE, GERD, and controls. A, EDN at baseline; bars show median with IQR. B, EDN after treatment initiation in EoE patients (Wilcoxon test). C, ECP at baseline; bars show median with IQR. D, ECP after treatment initiation in EoE patients (paired *t* test). E, TARC at baseline Bars show mean and SD. ANOVA *P*=0.434. F, TARC after treatment initiation in EoE patients (calculated by paired *t* test).

Next, we sought to examine eosinophil-specific proteins to correlate and compare these biomarkers with the elevated FeNO and AEC in EoE. Serum EDN was significantly higher in patients with active EoE compared with control individuals (median 72.36 ng/mL vs. 26.4 ng/mL, *P*=0.009) and EoE patients in remission (median EDN 23.41 ng/mL, *P*=0.006) but did not differ compared with patients with GERD (median 42.45 ng/mL, *P*=0.218, Fig. 4A). In a ROC-analysis, a cutoff for serum EDN of 50.73 ng/mL had a sensitivity of 75.8% and specificity of 73.3% to discriminate between active EoE and non-EoE (GERD and control individuals; Supplemental Fig. 1C, Supplemental Digital Content 1, http://links.lww.com/JCG/B129). In treatment responders, serum EDN significantly decreased (median EDN at baseline 67.4 ng/mL vs. 11.96 ng/mL, *P*=0.002), whereas in nonresponders no difference in serum EDN levels (median EDN at baseline 63.51 vs. 52.95 ng/mL, *P*=0.578, Fig. 4B) was observed.

Conversely, we found no difference in fecal EDN between the study groups or upon treatment, regardless of the therapeutic response or failure (Supplemental Figs. 2A and 2B, Supplemental Digital Content 1, http://links.lww.com/JCG/B129). One patient with GERD and one control individual were colonized with *Helicobacter pylori* and therefore excluded from stool analysis.

### Increased ECP in Active EoE With Decrease in Treatment Responders

Serum ECP was significantly increased in active EoE compared with control individuals (median serum ECP 112.3 vs. 56.3 ng/mL, *P*=0.009). However, there was no difference compared with GERD patients (median serum ECP 136 ng/mL, *P*=0.99) or EoE patients in remission (median serum ECP 76.65 ng/mL, *P*=0.14, Fig. 4C). Upon therapy, a significant decrease in serum ECP was observed in responders (mean serum ECP 135.3 vs. 65.5 ng/mL, *P*=0.006) while no reduction was observed in nonresponders (mean 112 vs. 136.6 ng/mL, *P*=0.551; Fig. 4D).

Levels of fecal ECP did not differ between active EoE, GERD patients and control individuals. Median fecal ECP in active EoE was 907.4 ng/g stool versus a median of 684 ng/g in GERD, *P*=0.99, and 713.7 ng/g in control individuals, *P*=0.99. Furthermore, there was no difference in fecal ECP in treatment responders (median ECP 1628 ng/g vs. 591.6 ng/g, *P*=0.275) as well as in nonresponders (median ECP 330.7 ng/g vs. 848.8 ng/g stool, *P*=0.313) compared with baseline (Supplemental Figs. 2C and 2D, Supplemental Digital Content 1, http://links.lww.com/JCG/B129).

### Decrease of Thymus and Activation of Regulated Chemokine (TARC) upon Treatment Response

Serum TARC measurements showed no difference between patients with active EoE, control individuals, GERD, and EoE patients in remission (mean TARC level 833.2 vs. 783.3 pg/mL, *P*=0.93 vs. 1049 pg/mL, *P*=0.45 vs. 807.5 pg/mL, *P*=0.93 respectively, Fig. 4E). Interestingly, we observed a significant decrease of TARC during treatment in EoE patients with treatment response (mean TARC levels pretreatment 913.4 vs. 686.8 pg/mL post-treatment, *P*=0.027). Conversely, TARC levels did not decrease in nonresponders (mean TARC pretreatment 871.3 vs. 1063 pg/mL, *P*=0.068, Fig. 4F). TARC was not detectable in most fecal samples and, therefore not further assessed.

### No Elevation of Major Basic Protein 1 (MBP-1) in EoE

Serum MBP-1 did not differ between active EoE and GERD (mean MBP-1 526.2 vs. 574.7 ng/mL, *P*=0.761) and was not elevated compared with control individuals (mean MBP1 526.2 vs. 464.5 ng/mL, *P*=0.75) or EoE patients in remission (mean MBP1 569.2 ng/mL, *P*=0.761, Supplemental Fig. 3A, Supplemental Digital Content 1, http://links.lww.com/JCG/B129). Furthermore, MBP-1 showed no decrease in treatment responders and nonresponders (Supplemental Fig. 3B, Supplemental Digital Content 1, http://links.lww.com/JCG/B129). Like TARC, MBP-1 was not detectable in most fecal samples and therefore not further assessed.

### No Elevation of Fecal Calprotectin (fCal) in EoE

Surprisingly, fCal levels significantly increased in control individuals compared with patients with active EoE (median 73.1 stool vs. 12.4 μg/g, *P*=0.024, Supplemental Fig. 2E, Supplemental Digital Content 1, http://links.lww.com/JCG/B129). No difference was observed between patients with active EoE, EoE in remission, and GERD (median 12.4 vs. 14.8 μg/g stool, *P*=0.854 vs. 32.4 ug/g, *P*=0.18). Furthermore, regardless of the treatment response in EoE, no difference in fCal was observed (Supplemental Fig. 2F, Supplemental Digital Content 1, http://links.lww.com/JCG/B129). Many patients had no detectable fCal values, and results should be interpreted cautiously.

### FeNO Correlates With AEC and Serum EDN in Active EoE

**FIGURE 5:**
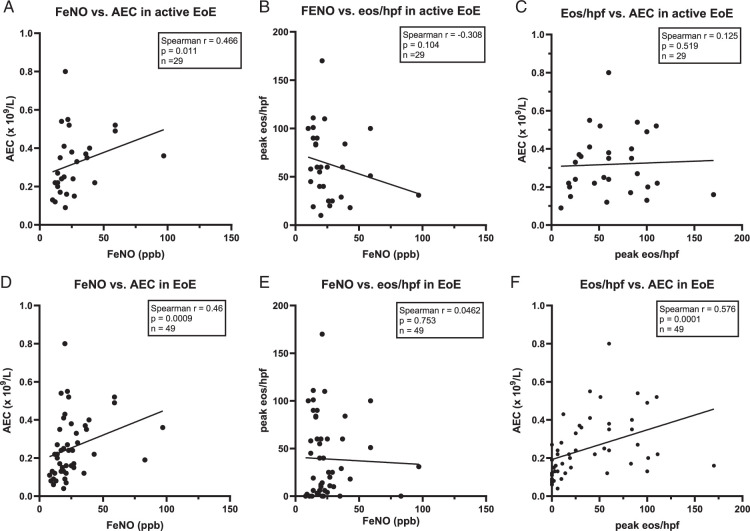
Scatterplots of biomarker correlations in EoE. Calculation with the Spearman nonparametric correlation. Simple linear regression is shown in graphs. A, Correlation of FeNO versus AEC in active EoE. B, Correlation of FeNO versus eos/hpf in active EoE. C, Correlation of eos/hpf versus AEC in active EoE. D, Correlation of FeNO versus AEC in all EoE patients (active EoE and EoE in remission). E, Correlation of FeNO versus eos/hpf in all EoE patients. F, Correlation of eos/hpf versus AEC in all EoE patients.

Next, we examined the correlation of FeNO to other biomarkers. Focusing on active EoE, we observed a moderate but significant correlation between FeNO and AEC (Spearman r=0.466, *P*=0.011, Fig. 5A). However, there was no significant correlation between FeNO and esophageal eosinophil infiltration (number of eos/hpf; r=−0.308, *P*=0.104; Fig. 5B), mEREFS, age or time of symptom onset in patients with active EoE. Furthermore, there was no significant correlation between eos/hpf and AEC in patients with active EoE (Spearman r=0.125, *P*=0.52, Fig. 5C). Interestingly, serum EDN was the only serum biomarker correlating with FeNO (Spearman rank of 0.463, *P*=0.012; Supplementary Figure 4A, Supplemental Digital Content 1, http://links.lww.com/JCG/B129). Analysis of all EoE patients showed a correlation between FeNO and AEC and between eos/hpf versus AEC but not FeNO vs. eos/hpf (Figs. 5D–F).

Further analysis in active EoE revealed a strong correlation between serum EDN and AEC (Spearman r=0.75, *P*<0.0001). However, no significant correlation between EDN and eos/hpf was observed (Spearman r=0.019, *P*=0.92, Supplemental Figs. 4B, C, Supplemental Digital Content 1, http://links.lww.com/JCG/B129). In addition, serum ECP showed a moderate correlation with AEC (Spearman r=0.493, *P*=0.007) but not with eos/hpf and FeNO (Spearman r=0.039, *P*=0.84 and Spearman r=0.23, *P*=0.23 respectively, Supplemental Figs. 5A–C, Supplemental Digital Content 1, http://links.lww.com/JCG/B129). None of the other markers examined, including fCal, significantly correlated with age, years since symptom onset, mEREFS, eos/hpf, AEC, and FeNO. Correlating the serum markers with each other, only EDN and ECP showed a high degree of correlation (Spearman r=0.587, *P*=0.0008, Supplemental Fig. 5D, Supplemental Digital Content 1, http://links.lww.com/JCG/B129).

## DISCUSSION

Assessment of FeNO is an established clinical procedure for pulmonary patients suffering from asthma.^18,19^ As asthma, EoE is a Th2-driven disease. Therefore, we hypothesized that FeNO could be a potential marker for the diagnosis of EoE and a suitable marker for monitoring disease activity. Patients with physician-diagnosed asthma or regular inhaler use were excluded to eliminate the interference of a concomitant obstructive pulmonary disease on FeNO measurements. Lung function testing with body plethysmography was performed in all patients to identify functional airway obstruction. Our stringent study protocol omitted the possibility that concomitant undiagnosed obstructive pulmonary disease, presumably Th2-driven bronchial asthma, influenced the FeNO measurements. Our study showed that FeNO levels are significantly higher in patients with active EoE compared with GERD and control individuals. However, FeNO was not helpful in monitoring disease activity during the treatment, as it was in line with previous studies using smaller cohorts with less stringent inclusion criteria, mainly focusing on children.^32–35^ We included GERD as an additional group and provided the largest adult EoE cohort to show that FeNO differentiates between active EoE and GERD.

Leung et al^35^ reported significantly lower median FeNO levels after treatment (20.3 vs. 17.6 ppb) in 11 patients with EoE that did not predict a histologic response potentially explained by not discriminating between treatment responders and nonresponders. In our analysis, the median FeNO reduction after treatment was −26.7% in responders compared with −18.7% in nonresponders and did not reach statistical significance, unlike Leung et al^35^ After distinguishing treatment responders from nonresponders, subanalysis did not show significantly reduced FeNO levels in treatment responders. Interestingly, patients with active EoE showed a moderate but significant correlation between FeNO and peripheral AEC. However, there was no correlation between FeNO levels and peak eos/hpf in the esophageal biopsies. In light of these findings, FeNO is not a suitable marker for monitoring disease activity in EoE.

On the contrary, analysis of AEC, ECP, and EDN may help to diagnose and monitor patients with EoE. Several studies have targeted eosinophil-specific proteins as potential biomarkers for diagnosis and disease monitoring.^27,28,36–44^ In a systematic review of Radonjic et al,^45^ eotaxin-3, TARC, AEC, ECP, and mast cell tryptase were identified as potential candidates to discriminate between active EoE and EoE in remission after treatment. In line with previous studies, we observed that AEC and the eosinophil-specific proteins ECP and EDN are increased in active EoE. In addition, a significant decrease in AEC, ECP, EDN, and TARC was observed in treatment responders. Even more, EDN could discriminate active EoE patients from patients in remission. However, none of these markers showed a significant correlation with the number of eos/hpf which is currently the most important diagnostic criterium for assessment of inflammatory activity in the esophageal mucosa. Hence, the predictive value of these markers remains uncertain. Nevertheless, our results suggest that AEC, EDN, and ECP may help to discriminate between treatment responders and nonresponders. Unfortunately, we did not reach sufficient power to establish an absolute cutoff value for each of these.

Fecal biomarkers have been extensively studied in lower gastrointestinal tract diseases such as IBD, irritable bowel syndrome (IBS), allergic colitis, and collagenous colitis.^29,30,46–49^ Theoretically, proteins released by eosinophils in the esophagus during active inflammation may transit through the lower gastrointestinal tract and be excreted in the patient’s stool. Hence, they could serve as a simple, cost-effective, and easy-to-collect option for disease monitoring. With only 2 published studies assessing fecal EDN, there is little evidence for fecal biomarkers in EoE.^40,44^ To our knowledge, no other evaluations of fecal parameters as noninvasive biomarkers in EoE have been performed. Here, we investigated fecal ECP, EDN, and calprotectin as potential biomarkers for EoE. Fecal calprotectin—routinely used for disease monitoring in IBD—did not show consistent elevation in patients with EoE, and some control individuals displayed higher fCal levels than EoE patients. The elevation of fCal levels in control individuals might be caused by the underlying gastrointestinal symptoms that qualified them for an EGD examination. Furthermore, fCal levels can also be increased due to pathologies of the small intestine or the lower gastrointestinal tract, which were not excluded by imaging or colonoscopy in our study. Therefore, neutrophil granulocyte-derived fCal seems not to be a suitable marker for disease monitoring in patients with EoE. Fecal ECP was slightly elevated in patients with active EoE. However, fecal ECP levels did not change throughout treatment. Fecal EDN, similar to fCal, did not show a difference between study groups and was undetectable in many patients. Furthermore, none of the examined fecal biomarkers correlated with the number of eosinophils in esophageal biopsies and, therefore, do not seem suitable for disease monitoring in EoE. However, large-scale cohort studies need to confirm these findings to evaluate whether fecal biomarkers, especially fecal ECP and fecal EDN, are indeed unsuitable as biomarkers for diagnosis or disease monitoring in EoE.

Our study had several limitations. Due to the observational, noninvasive nature of the study, the time intervals between baseline and follow-up gastroscopy with biomarker assessment after initiation of a new treatment varied from 6 weeks up to 1 year, and the patient’s choice for a treatment led to different treatments at follow-up (dietary therapy, PPI, or topical steroids). Nevertheless, a biomarker should provide reliable information on disease activity at different time points of the disease course and independent of the chosen treatment regimen. Another limitation was the small number of follow-up patients in remission after treatment initiation (11 responders, 7 nonresponders). However, the simultaneous assessment of FeNO, blood and fecal biomarkers in our cohort strengthens our findings on potential biomarkers in patients with EoE. Moreover, the overall adherence to the study protocol was good, with no dropouts and patients consenting to continuous follow-up biomarker level measurements.

## CONCLUSIONS

In conclusion, our study suggests that FeNO may have diagnostic value in differentiating patients with active EoE from non-EoE patients. However, it does not seem to be a suitable marker for disease monitoring in patients with EoE. Interestingly, AEC and the eosinophil-specific proteins EDN, ECP, and TARC decreased with treatment response. Therefore, larger cohort studies are needed to prove the clinical value of these promising candidates for disease monitoring in EoE.

## Supplementary Material
