# Supplementary material for: Noninvasive Disease Assessment in Eosinophilic Esophagitis With Fractionated Exhaled Nitric Oxide, Blood, and Fecal Biomarkers
Source: J Clin Gastroenterol. 2024 Sep 2;59(8):725–36. doi: 10.1097/MCG.0000000000002068 (PMC12329813; doi:10.1097/MCG.0000000000002068)
Supplement: Supplementary file 1 [file mcg-59-725-s001.docx]

**Supplemental Table 1**

| Body plethysmography | EoE n = 38 | GERD n = 14 | Controls n = 17 | p-value |
| --- | --- | --- | --- | --- |
| FEV 1 (l) | 3.73 ± 0.819 | 3.59 ± 0.79 | 3.45 ± 0.62 | 0.439 |
| FEV 1 % of prediction (%) | 97.39 ± 11.06 | 98.6 ± 12.6 | 94.1 ± 10 | 0.473 |
| FVC (l) | 4.59 ± 1.06 | 4.42 ± 1.07 | 4.27 ± 0.74 | 0.547 |
| FVC % of prediction (%) | 96.32 ± 11.37 | 97.8 ± 13.1 | 94.2 ± 7.5 | 0.659 |
| FEV1 % FVC (%) = Tiffeneau | 81.6 ± 5.93 | 81.7 ± 4.5 | 80.6 ± 3.55 | 0.782 |
| DLCO_SB (mmol/(min x kPa)) | 10.06 ± 2.14 | 9.18 ± 1.83 | 9.11 ± 1.87 | 0.175 |
| TLC (l) | 6.55 ± 1.33 | 6.18 ± 1.36 | 6.28 ± 0.91 | 0.564 |
| TLC % of prediction (%) | 99.45 ± 11.09 | 97.4 ± 11 | 101 ± 9.84 | 0.631 |

**Supplemental Figure 1**
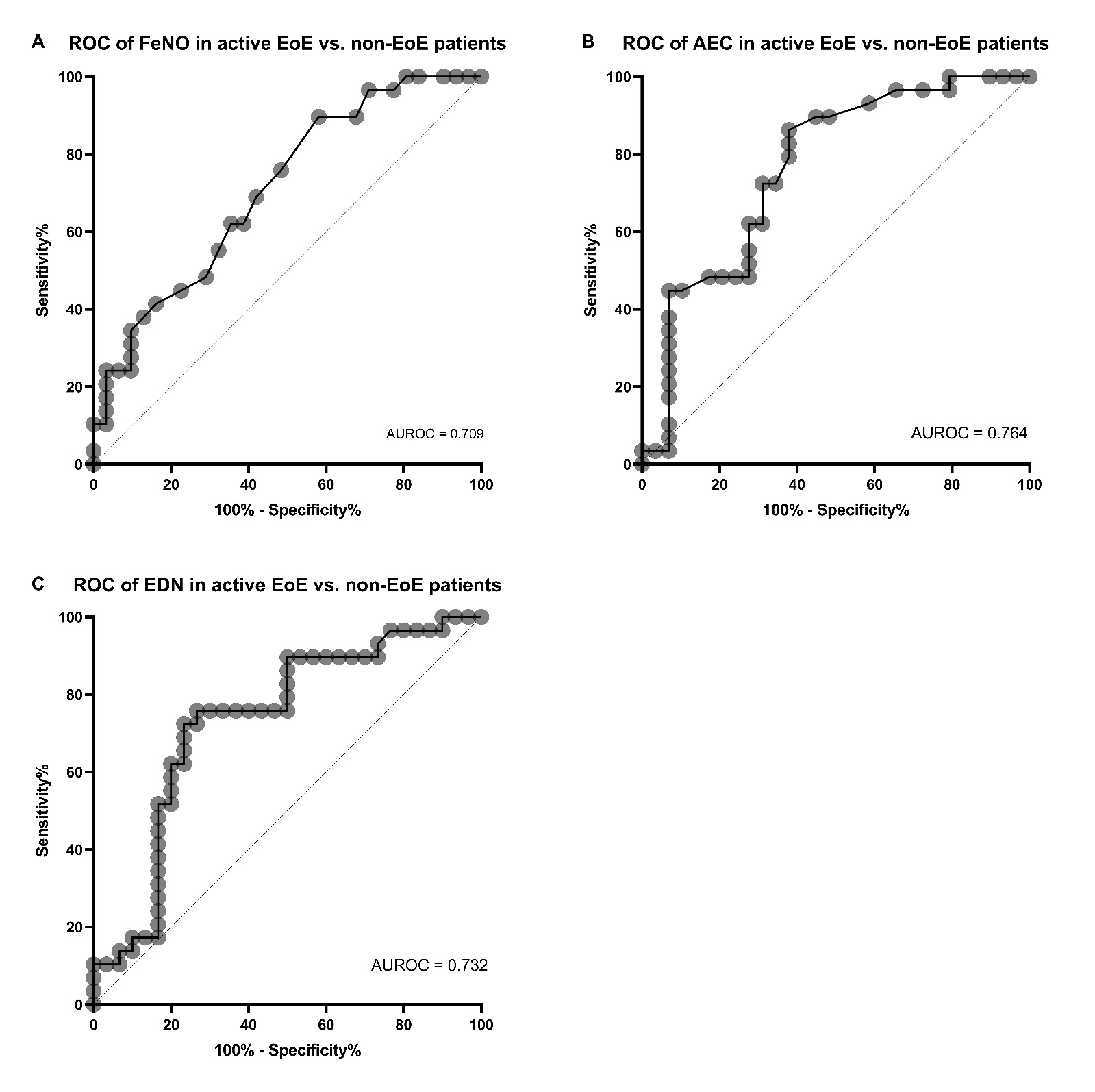


**Supplemental Figure 2**
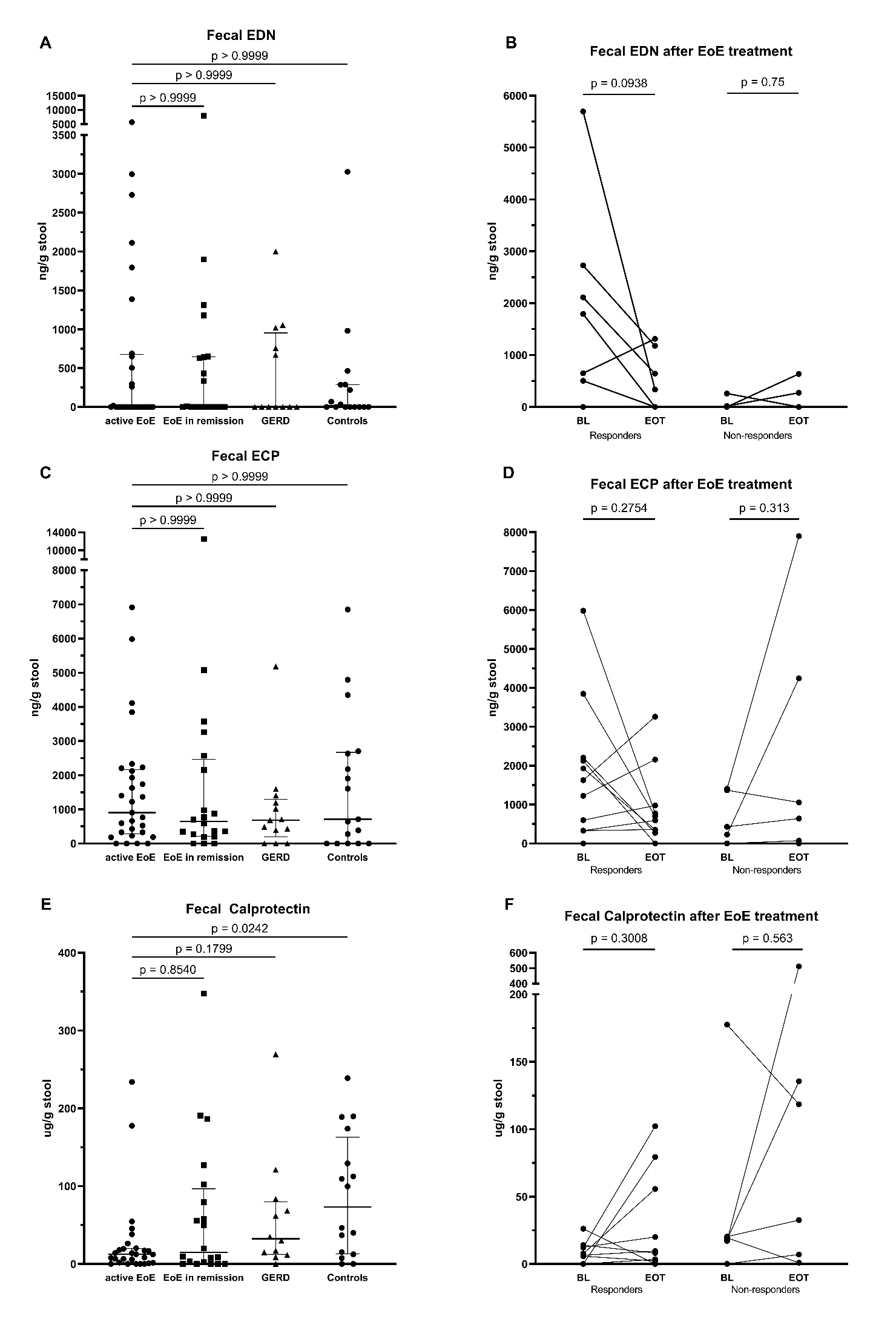


**Supplemental Figure 3**
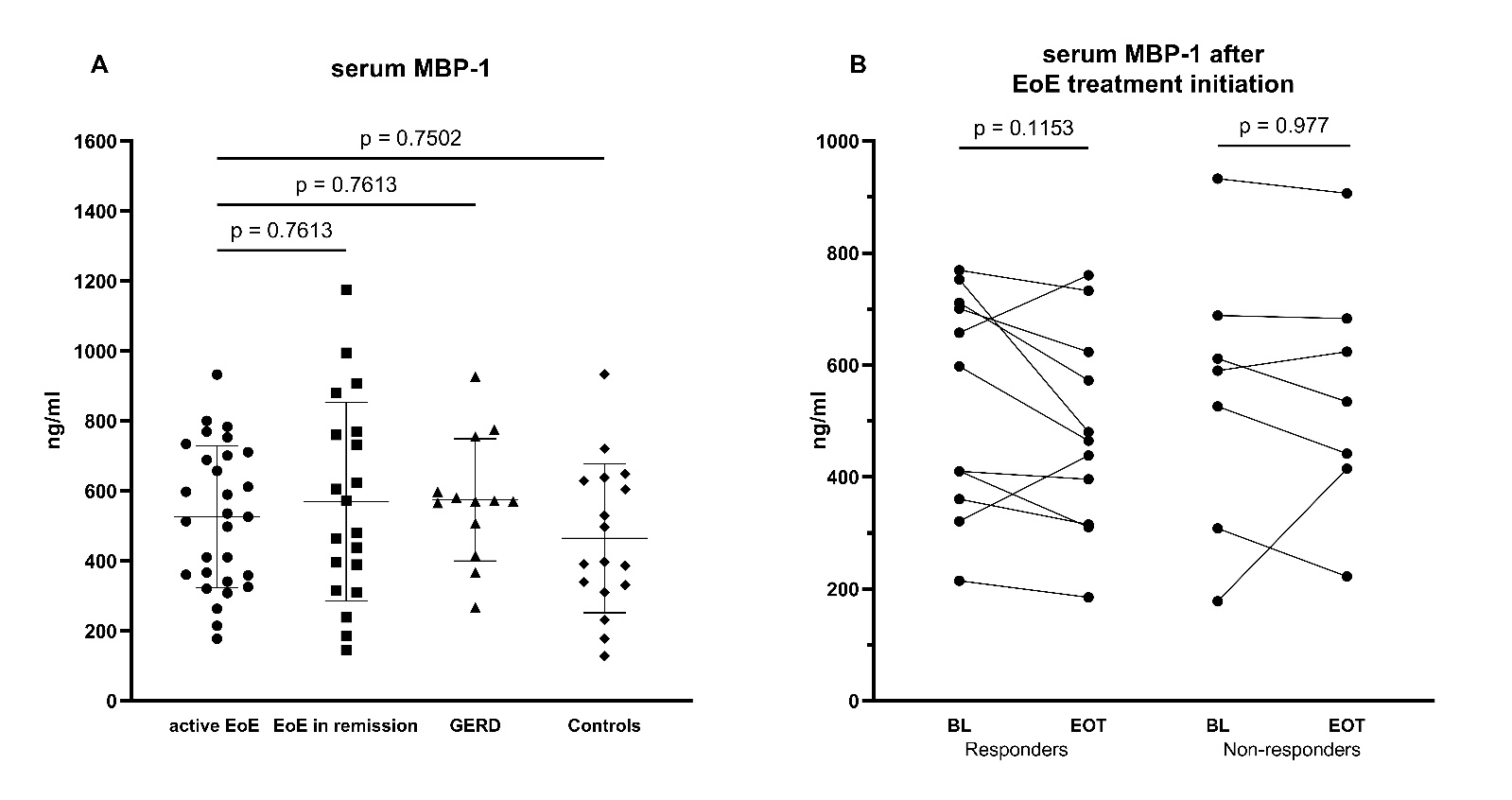


**Supplemental Figure 4**
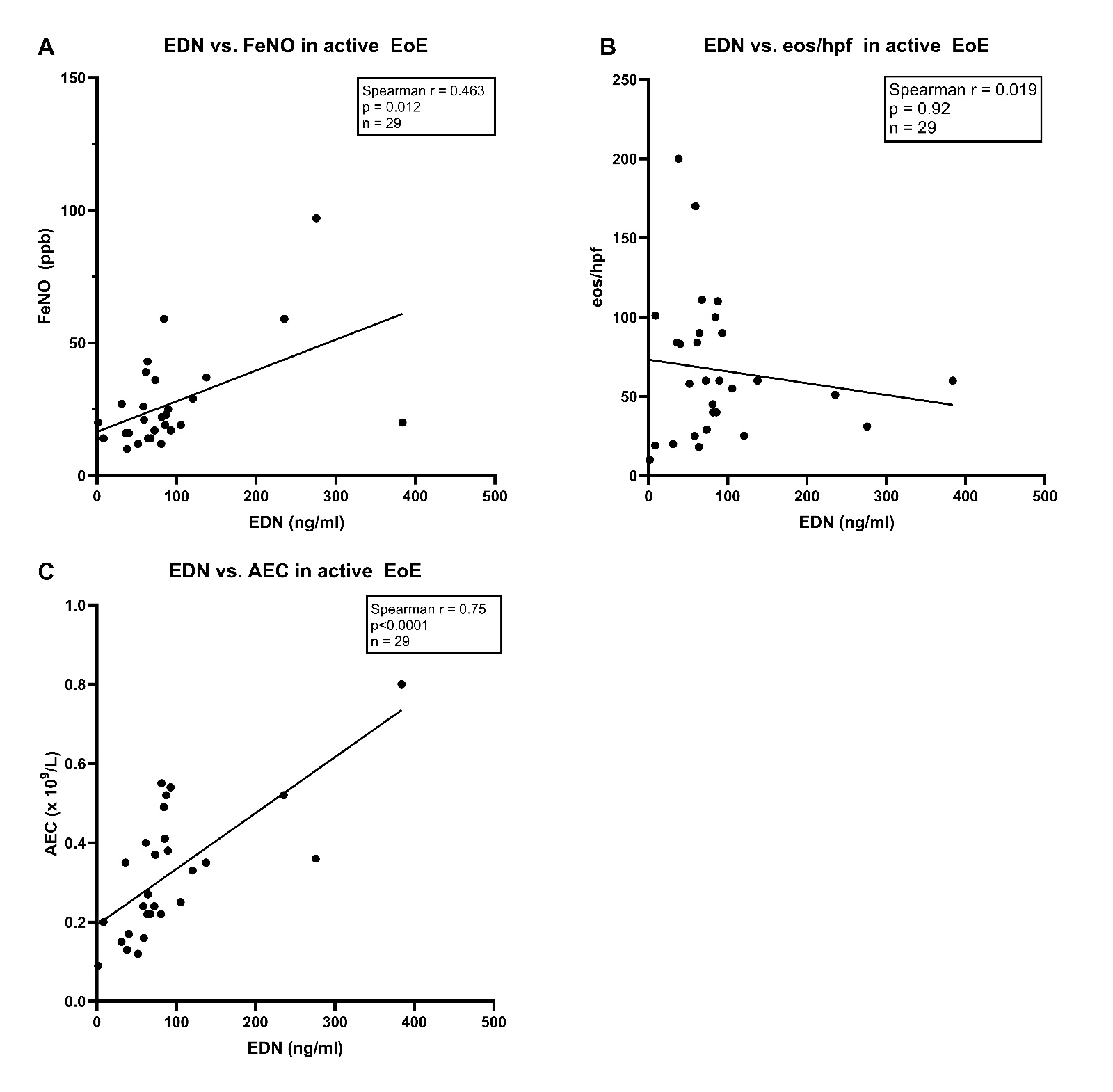


**Supplemental Figure 5**
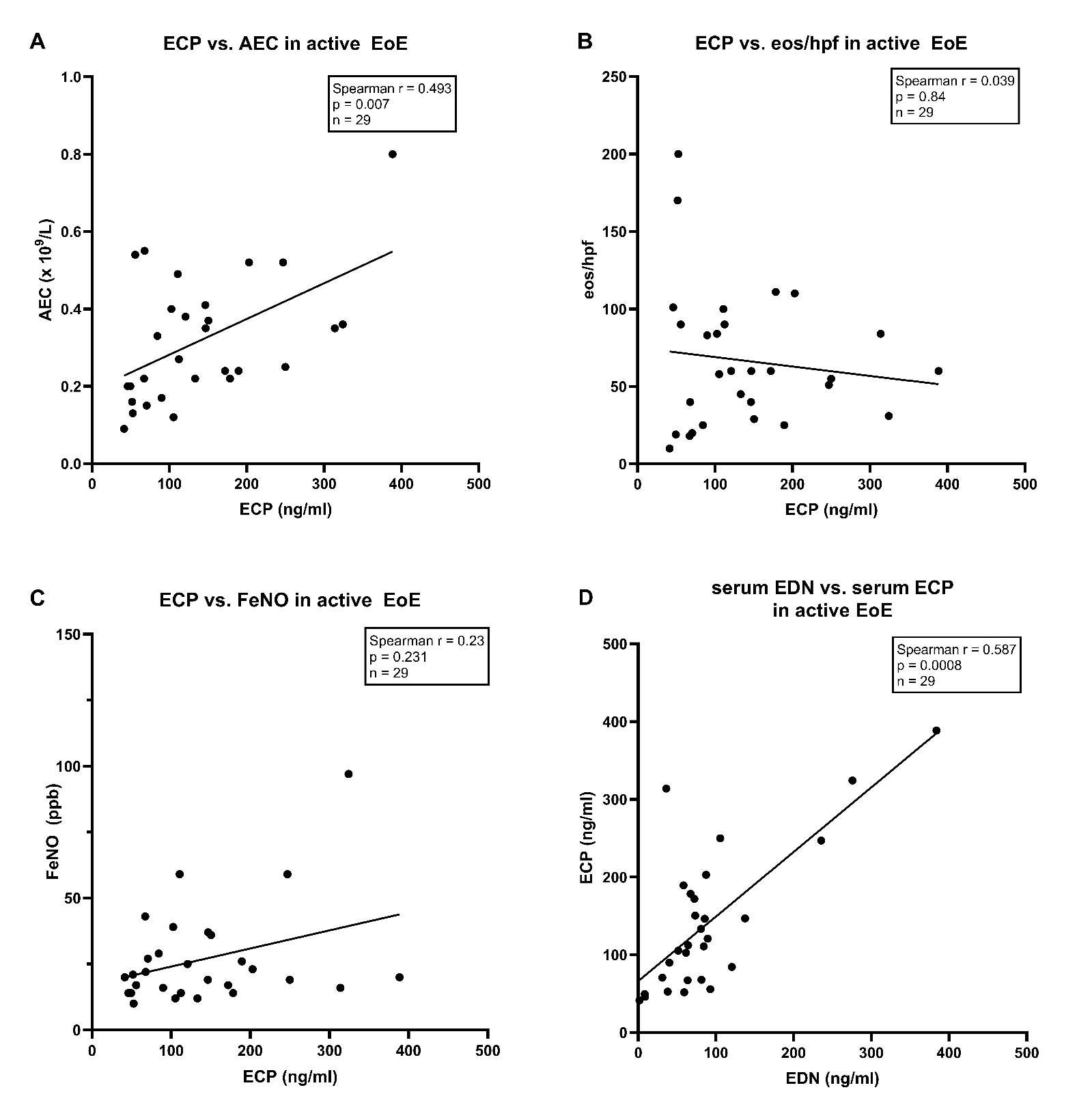


**Supplemental figures and table legends**

**Supplemental Table 1:** Group comparison was done with ordinary one-way ANOVA testing. FEV1 = forced expiratory flow in the first second of forced expiration, FVC = forced vital capacity, DLCO_SB = single-breath diffusing capacity of the lung for CO, TLC = total lung capacity. Values are shown as mean and standard deviation, Tiffeneau is shown as percentage.

**Supplemental Figure 1:** Receiver operator curves (ROC) in patients with active EoE vs. non - EoE patients (GERD and control individuals). **1A:** ROC analysis of FeNO in active EoE vs. non-EoE patients. Area under the ROC curve (AUROC) = 0.709. **1B:** ROC analysis of AEC in active EoE vs. non-EoE patients. Area under the ROC curve = 0.764. **1C:** ROC analysis of serum EDN in active EoE vs. non-EoE patients. Area under the ROC curve = 0.732.

**Supplemental Figure 2:** Fecal biomarker levels. Bars show median and IQR. **2A**: Fecal EDN in patients with EoE compared to non-EoE patients. **2B:** Fecal EDN before and after EoE treatment initiation. **2C**: Fecal ECP in patients with EoE compared to non-EoE patients. **2D**: Fecal ECP before and after EoE treatment initiation. **2E:** Fecal calprotectin in EoE compared to non-EoE patients. **2F**: Fecal calprotectin before and after EoE treatment initiation.

**Supplemental Figure 3:** Serum major basic protein 1 (MBP-1) levels. **3A:** Serum MBP-1 levels in EoE vs. non - EoE patients. Bars show mean and SD. **3B:** Serum MBP-1 in EoE patients before and after treatment initiation (calculated by paired t-test).

**Supplemental Figure 4:** Correlations of serum EDN vs. other biomarkers in active EoE. Correlations were calculated with the Spearman nonparametric correlation. Simple linear regression is shown in graphs. **4A**: Correlation of serum EDN vs. FeNO**.** **4B**: Correlation of serum EDN vs. eos/hpf. **4C**: Correlation of serum EDN vs. AEC in active EoE.

**Supplemental Figure 5:** Correlation of different biomarkers in active EoE. Correlations were calculated with the Spearman nonparametric correlation. Simple linear regression is shown in graphs **5A:** Correlation of serum ECP vs. AEC. **5B:** Correlation of serum ECP vs. eos/hpf. **5C:** Correlation of serum ECP vs. FeNO. **5D:** Correlation of serum EDN vs. serum ECP.
